# Multidrug-Resistant Tuberculosis in Admitted Patients at a Tertiary Referral Hospital of Bangladesh

**DOI:** 10.1371/journal.pone.0040545

**Published:** 2012-07-11

**Authors:** Sayera Banu, Asif Mujtaba Mahmud, Md. Toufiq Rahman, Arman Hossain, Mohammad Khaja Mafij Uddin, Tahmeed Ahmed, Razia Khatun, Wahiduzzaman Akhanda, Roland Brosch

**Affiliations:** 1 International Centre for Diarrhoeal Disease Research, Bangladesh, Dhaka, Bangladesh; 2 National Institute of Diseases of Chest and Hospital, Dhaka, Bangladesh; 3 Institut Pasteur, Integrated Mycobacterial Pathogenomics Unit, Paris, France; McGill University, Canada

## Abstract

**Background:**

This study was set out to investigate the magnitude, patterns and molecular characterization of drug-resistant *Mycobacterium tuberculosis* strains at a tertiary referral hospital in Bangladesh.

**Methods:**

Pulmonary tuberculosis (TB) patients admitted at National Institute of Diseases of the Chest and Hospital from February 2002 to September 2005 with or without previous history of TB and/or other complications were randomly interviewed. Among 265 participants enrolled, *M. tuberculosis* isolates from 189 patients were finally tested for susceptibility to rifampicin (RMP), isoniazid (INH), ethambutol (ETM) and streptomycin (STM). Genotyping of *M. tuberculosis* was done using deletion analysis and spoligotyping.

**Results:**

Eighty-eight percent (n = 167) of the patients had history of previous anti-TB treatment while the remaining 12% were new TB cases. Of the 189 isolates, 9% were fully susceptible to the first line anti-TB drugs and 73.5% were multi-drug resistant TB. Other susceptibility results showed 79.4%, 77.2%, 76.7% and 78.8% resistance to INH, RMP, ETM and STM respectively. Multi-drug resistance was significantly higher among the 130 (78%) patients with previous history of anti-tuberculosis treatment (95% confidence interval, p = 0.001). Among the 189 analyzed isolates, 69% were classified as “modern” *M. tuberculosis* strains (i.e. TbD1- strains, lacking the *M. tuberculosis*-deletion region TbD1), whereas the remaining 31% were found to belong to the “ancestal” TbD1+ *M. tuberculosis* lineages. One hundred and five different spoligotype patterns were identified in which 16 clusters contained 100 strains and 89 strains had unique pattern. Strains with a spoligotype characteristic for the “Beijing” cluster were predominant (19%) and most of these strains (75%) were multi-drug resistant (MDR).

**Conclusions:**

A high level of drug resistance observed among the re-treatment patients poses a threat of transmission of resistant strains to susceptible persons in the community. Proper counseling of patients and attention towards the completion of the anti-TB treatment is needed.

## Introduction

Tuberculosis (TB) is a disease of global importance. The emergence of resistance to anti TB drugs, and particularly multidrug-resistant tuberculosis (MDR-TB), has been identified as one of the major obstacles to global TB control [1]. MDR-TB, defined as TB caused by organisms that are resistant to at least isoniazid (INH) and rifampicin (RMP), two most potent first-line anti-TB drugs, continues to further worsen the current global TB situation. Besides, advent of human immunodeficiency virus (HIV) has heightened this disease burden both in industrial and developing countries [2], [3]. World Health Organization (WHO) has estimated that over 500,000 cases of MDR-TB occur annually worldwide and two million people die of TB each year [4], [5].

TB is a major public health problem in Bangladesh. In 2008, the World Health Organization (WHO) ranked Bangladesh sixth among the world’s 22 high-burden TB countries and 9^th^ among 25 high priority MDR and extensively drug resistant (XDR) TB countries. In 2007, an estimated 353,103 new cases, 1,587,797 of which were sputum smear-positive (SS+) TB cases occurred and more than 70,900 TB-related deaths occurred. The TB mortality rate (45 deaths per 100,000 population) in Bangladesh is higher than the Southeast Asian region (31 deaths per 100,000 population).

Although Bangladesh is a low-HIV/AIDS-prevalence country, the National Tuberculosis Control Programme (NTP) is introducing more collaborative TB-HIV/AIDS-related activities as well as managing MDR TB. The level of drug resistance is considered as an epidemiological indicator to assess the achievement of the NTP, as well as it measures the extent of resistant bacteria prevailing in the community. However, data on the prevalence of drug resistant TB in Bangladesh are scarce and very limited surveillance exists for these strains. In the recent past, a community based survey in a rural and urban set up reported that among the identified TB cases about 48.4% of *M. tuberculosis* isolates were resistant to at least one of the first line anti-TB drugs while multidrug resistance was observed in 5.5% of isolates [6]. In the 1990s, primary anti tubercular drug resistance in a hospital based study in Dhaka was as follows: INH 16%,RMP 11%, streptomycin (STM) 7% and ethambutol (ETM) 4%. MDR TB was found in 5% cases [7]. A recent estimation made by WHO showed approximately 2.2% of new and 14.7% of previously treated patients suffer from MDR TB in Bangladesh [8].

The genotyping of *M. tuberculosis* strains is important for TB control because it allows the detection of suspected outbreaks and the tracing of transmission chains. It is also important to monitor species diversity, as well as to identify secondary infections [9].

Deletion analysis, a PCR-based technique, has been shown to be more effective in differentiating members of the *M. tuberculosis* complex as well as larger phylogenetic clusters within *M. tuberculosis* and is based on the presence or absence of 20 regions of difference (RD) in the genomes of members of *M. tuberculosis* complex [9], [10]. In particular, *M. tuberculosis* can be divided into “ancestral” and “modern”lineages based on *M. tuberculosis*-specific deletion region 1 (TbD1) that is present in ancestral, TbD1+ *M. tuberculosis* strains and is absent in modern, TbD1- *M. tuberculosis* strain.

Spoligotyping is a PCR-based method, which allows simultaneous detection and strain differentiation of *M. tuberculosis* present in clinical specimens without the need for culture. Spoligotyping is based on the polymorphism in the direct repeat (DR) locus of the mycobacterial chromosome. The DR locus is one of the most well studied loci of the *M. tuberculosis* genome showing considerable strain-to-strain polymorphisms [11]. The DR locus comprises a series of 36-bp DR elements interspersed with unique spacer sequences, which vary in size from 35 to 41 bp [12]. Clinical isolates can be differentiated by the presence or absence of one or more spacers.

The objective of the study was to investigate the magnitude, pattern of drug resistance as well as to provide the molecular characterization of the strains responsible for drug resistant pulmonary tuberculosis (PTB) for further comparison and strain tracing among the patients of a reference hospital, the National Institute of Diseases of the Chest and Hospital (NIDCH), Dhaka.

## Materials and Methods

**Figure 1:**
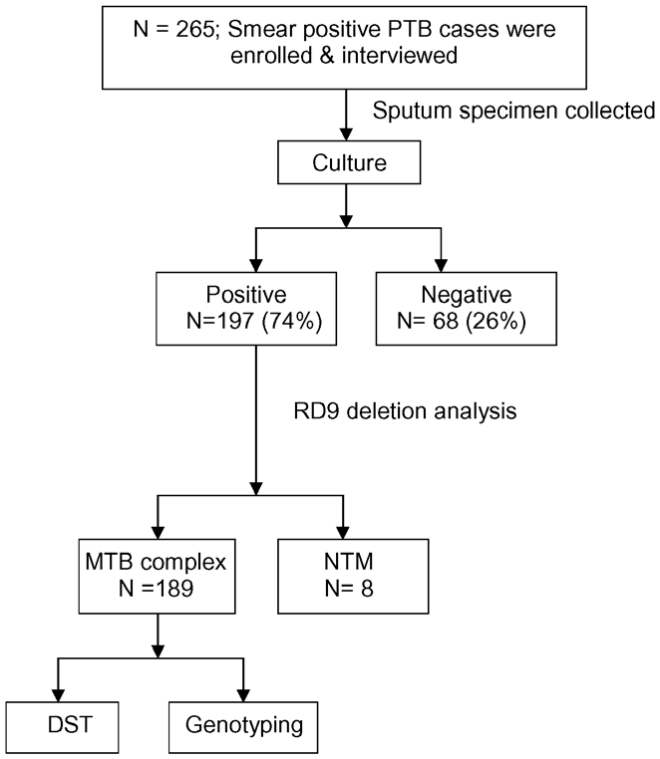
Flow diagram of tuberculosis patient at enrolment.

**Table 1:** Distribution of ‘ancestral’ and ‘modern’ *M. tuberculosis* strains within divisions.

Division	Dhaka (%)	Barisal (%)	Khulna (%)	Sylhet (%)	Chittagong (%)	Rajshahi (%)	Rangpur (%)	Total (%)
**Ancestral**	33 (39.3)	02 (11.1)	03 (27.3)	06 (85.7)	08(20.5)	03(33.3)	01(33.3)	56 (32.7)
**Modern**	51 (60.7)	16 (88.9)	08 (72.7)	01 (14.3)	31(79.5)	05(66.7)	2(66.7)	115 (67.3)
**Total (%)**	84(49.1)	18 (10.5)	11 (6.4)	7 (4.1)	39 (22.8)	9 (5.3)	3 (1.8)	171(100)

**Figure 2:**
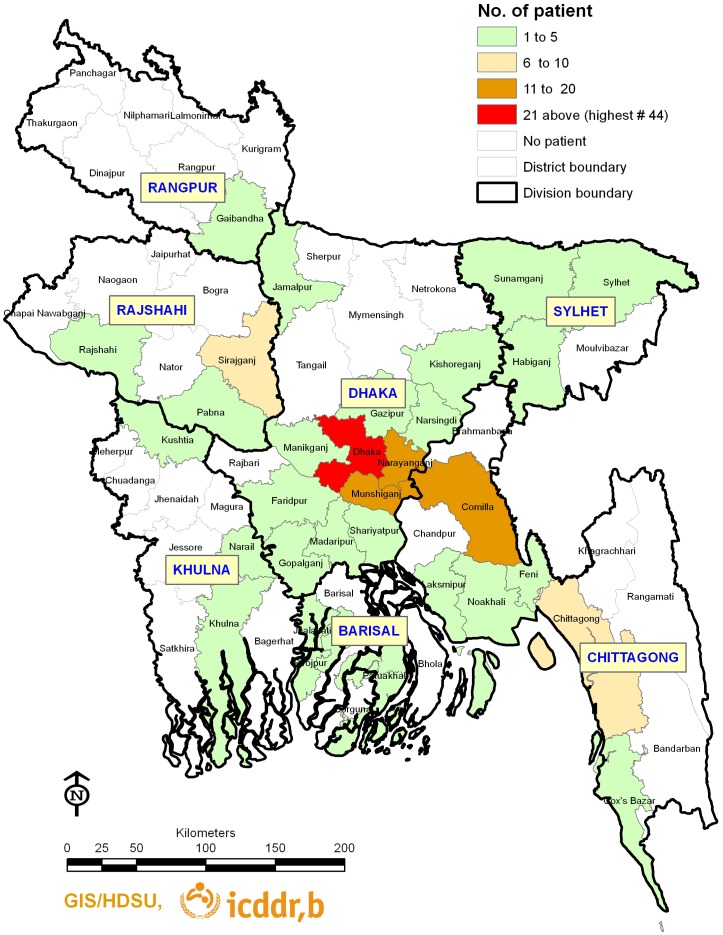
Map of Bangladesh showing the locations of TB cases.

The study protocol was reviewed and approved by the Research Review Committee and the Ethics Review Committee of the International Centre for Diarrhoeal Disease Research, Bangladesh (icddr,b). The participants were enrolled into the study only after receiving informed written consent from themselves or from their parents/guardians if they were child (<18 years of age). An assent was also obtained from participants between 11–18 years of age.

The study was carried out at the inpatient department of NIDCH, the only tertiary referral hospital for TB and chest disease in Bangladesh, located in the central part of Dhaka Metropolitan City. Patients from all over the country mostly as referral cases attend this health facility. According to the national TB control guideline suspected MDR-TB cases from all over the country usually referred to this health facility [13]. Thus, the patients visiting this health facility can give an idea of the MDR-TB situation of the country. The study subjects were grouped according to their geographical locations. As it is a government-subsidized hospital, most of its patients are of low socioeconomic status.

**Table 2:** Socio demographic characteristics of TB patients.

Characteristics	Subcategory	number (%)
Mean age (yrs)		32.8
Sex (n = 189)	Male	142 (75.1)
	Female	47 (24.9)
Smoking (n = 173)	Yes	90 (52.0)
	No	83 (48.0)
Area (n = 171)	Rural	96 (56.1)
	Urban	75 (43.9)
Age (n = 189)	<45 yrs	148 (78.3)
	≥45 yrs	41 (21.7)
Occupation (n = 166)	Unemployed	24 (14.5)
	Self employed	74 (44.6)
	Service	34 (20.5)
	Business	10 (6.0)
	Housewife	24 (14.5)
Income (n = 120)	≤3000 TK	84 (70)
	>3000 TK	36 (30)
Educational status (n = 175)	Illiterate	39 (22.3)
	Secondary	114 (65.1)
	Higher	22 (12.6)
History of TB contact (n = 179)	Yes	61 (34.1)
	No	118 (65.9)
Presence of TB patient in family (n = 171)	Yes	17 (9.9)
	No	154 (90.1)
Previous history of TB Rx (n = 161)	Completed	66 (41)
	Not Completed	95 (59)

We randomly interviewed and enrolled the cases from the PTB patients admitted during the study period of February 2002 to September 2005 at NIDCH with or without previous history of TB and/or other complications like haemoptysis, pleural effusion, cachexia, anti-TB drug intolerance, ailing patients etc. We included all Category-II failure cases, every 5th patient with Category-I failure and every 20th patient with TB and other associated complications. The patients were diagnosed as having PTB on the basis of presenting symptoms, clinical signs, two acid-fast bacillus (AFB) smear-positive sputum samples and chest X-ray, if necessary. The patients were being treated with recommended standardized treatment regimen according to the patient category following the national TB control guidelines.

**Table 3:** Drug susceptibility pattern for *M. tuberculosis* clinical isolates.

Susceptibility Pattern	Sub category	Total patients (%)	New patients (%)	Previously treated patients (%)
		n = 189	n = 22	n = 167
**Fully susceptible**		17 (8.9)	03 (13.6)	14 (8.4)
**Any resistance**				
	Any STM	149 (78.8)	15 (68.2)	134 (80.2)
	Any INH	150 (79.4 )	12 (54.5)	138 (82.6)
	Any RMP	146 (77.2)	11 (50.0)	135 (80.8)
	Any ETM	145 (76.7 )	10 (45.5)	135 (80.8)
**Mono resistance**				
	Mono STM	10 (5.3)	4 (18.2)	6 (3.6)
	Mono INH	1 (0.5)	00	1 (4.5)
	Mono RMP	2 (1.1)	00	2 (1.2)
	Mono ETM	4 (2.1)	1 (4.5)	3 (1.8)
**Any two resistance**				
	STM + INH	3 (1.6)	1 (4.5)	2 (1.2)
	STM + RMP	1 (0.5)	1 (4.5)	00
	STM + ETM	1 (0.5)	00	1 (0.6)
	INH + RMP	4(2.1)	1(4.5)	3 (1.8)
	INH + ETM	2 (1.1)	00	2 (1.2)
	RMP + ETM	3 (1.6)	1 (4.5)	2 (1.2)
**Any three resistance**				
	STM + INH + RMP	6 (3.2)	1 (4.5)	5 (3.0)
	STM + INH + ETM	5 (2.6 )	1 (4.5)	4 (2.4)
	STM + RMP + ETM	1(0.5)	00	1 (0.6)
	INH + RMP + ETM	7 (3.7)	00	7 (4.2)
**All resistant**		122 (64.6)	7 (31.8)	115 (68.9)
**MDR**		139 (73.5)	9 (40.9)	130 (77.8)

The pulmonary TB patients were interviewed by a trained medical doctor using structured questionnaires. Clinical information including their demographic data, presenting signs and symptoms, history of prior diagnosis and treatment of TB, duration of previous anti-TB treatment, exposure to TB cases in the family and BCG vaccination were obtained from each patient. New TB patients were those cases who never had treatment for TB or who had taken anti-TB drugs for less than one month. Previously treated TB patients were patients who had received at least one month of anti-TB therapy. Patients who relapsed after having successfully completed treatment in the past, patients who failed treatment, patients who returned after interrupting treatment and chronic TB cases (had failed at least one retreatment course) were also included in this category. Cases of relapse, treatment failure, treatment interruption and chronic TB were identified according to the definition of WHO [14].

**Table 4:** Clusters and unique *M. tuberculosis* strains determined by spoligotyping method and distribution of spoligotyping-defined phylogenetic clades.

**No.** *	**Spoligotype pattern of isolate(s)**	**Octal code of spoligotype**	**Phylogenetic clade of isolate(s)** **
	**59 "Ancestral" ** ***M. tuberculosis*** ** strains (TbD1 region present)**		
5	▪▪▪▪▪▪▪▪▪▪▪▪▪▪▪▪▪▪▪▪▪▪▪▪▪▪▪▪□□□□▪□▪▪▪▪▪□□□□	777777777413700	EAI5 (138)
4	▪▪▪▪▪▪▪▪▪▪▪▪▪▪□▪▪▪▪▪▪▪▪▪▪▪▪▪□□□□▪□▪▪▪▪▪□▪▪▪	777767777413731	EAI1_SOM (1251)
3	▪▪▪▪▪▪▪▪▪▪▪▪▪▪□▪▪▪▪▪▪▪▪▪▪▪▪▪□□□□▪□▪▪▪▪▪□□□□	777767777413700	
3	▪▪▪▪▪▪▪▪▪▪▪▪▪▪▪▪▪▪▪▪▪▪▪▪▪▪▪▪□□□□▪□▪▪▪▪▪□▪▪▪	777777777413731	EAI1_SOM (48)
2	▪▪▪▪▪▪▪▪▪▪▪▪▪▪▪▪▪▪▪▪▪▪□▪▪▪▪▪□□□□▪□▪▪□▪▪▪▪▪▪	777777757413371	EAI undefined (292)
1	▪□□▪□□□▪▪□□□□▪□□□▪▪▪▪▪▪▪▪▪▪▪□□□□▪□▪▪▪▪▪▪▪▪▪	443021777413771	
1	▪▪▪▪▪▪▪▪▪▪▪▪▪▪▪▪▪▪▪▪▪▪□▪▪▪▪▪□□□□▪□▪▪▪▪▪□▪▪▪	777777757413731	
1	□□□▪▪▪▪▪▪▪▪▪▪▪□▪▪▪▪▪▪▪▪▪▪▪▪▪□□□□▪□▪▪▪▪▪□▪▪▪	077767777413731	EAI1_SOM (1734)
1	▪▪▪□□□▪□□□▪▪▪▪▪▪▪▪▪▪▪▪□▪▪▪▪▪□□□□▪□▪▪□□▪▪▪▪▪	704377757413171	
1	▪□□▪▪▪▪▪▪▪▪▪▪▪▪▪▪▪▪▪▪▪▪▪▪▪▪▪□□□□▪□▪▪□□□▪▪▪▪	477777777413071	EAI3_InD (11)
1	▪▪▪▪▪▪▪▪▪▪▪▪▪▪▪▪▪▪▪▪▪▪□▪▪▪▪□□□□□▪□▪▪□▪▪▪▪▪▪	777777757013371	
1	□▪▪▪▪▪▪▪▪▪▪▪▪▪▪▪▪▪▪▪▪▪▪▪▪▪▪▪□□□□▪□▪▪▪▪▪□□□□	377777777413700	
1	▪▪▪▪▪▪▪▪▪▪▪▪▪▪▪▪▪▪▪▪▪□□□□▪□□□□□□□□▪▪□▪▪▪▪▪▪	777777702003371	
1	▪▪▪▪▪▪▪▪▪▪▪▪▪▪▪▪▪▪▪▪▪▪□▪□▪□□□□□□□□▪▪□▪▪▪▪▪▪	777777752003371	
1	□□▪▪▪▪▪▪▪▪▪▪▪▪▪▪▪▪▪▪▪▪□▪▪▪▪▪□□□□▪□▪▪▪▪▪▪▪▪▪	177777757413771	
1	▪▪▪▪▪□□▪▪▪▪▪▪▪▪▪▪▪▪▪□□□□□▪▪▪□□□□▪□▪▪▪▪▪□□□□	763777603413700	
1	▪▪▪▪▪▪▪▪▪▪▪▪□□□▪▪▪▪▪▪▪▪▪▪▪▪▪□□□□▪□▪▪▪▪▪□▪▪▪	777707777413731	
1	▪▪▪▪▪▪□□▪▪▪▪▪▪□▪▪▪▪▪▪▪□□□□□□□□□□□□▪▪▪□□▪▪▪▪	771767740003471	
1	▪□▪▪▪▪▪▪▪▪▪▪▪▪□▪▪▪▪▪▪▪▪▪▪▪▪▪▪▪▪□□□▪▪▪▪▪▪▪▪▪	577767777743771	
1	▪▪▪▪▪▪▪▪▪▪▪▪▪▪□▪▪▪▪▪▪□□□□▪□□□□□□□□▪□□▪▪▪▪▪▪	777767702002371	
1	▪▪▪▪▪▪▪▪▪▪▪▪▪▪▪▪▪▪▪▪▪▪▪▪▪▪▪▪▪▪▪▪▪□▪▪▪▪▪□□□□	777777777773700	
1	▪▪▪▪▪▪▪▪▪▪□▪▪▪▪▪▪▪▪▪▪▪▪▪▪▪▪□□□□□□□▪▪▪▪▪□▪▪▪	777577777003731	EAI undefined (72)
1	▪▪▪▪▪▪▪▪▪▪▪▪▪▪▪▪▪▪▪▪▪▪▪▪▪▪▪▪▪▪▪▪▪□▪▪□▪▪▪▪▪▪	777777777773371	
1	▪□□▪▪▪▪▪▪▪▪▪▪▪□▪▪▪▪▪▪▪▪▪▪▪▪▪□□□□▪□▪▪□□□▪▪▪▪	477767777413071	
1	▪▪▪▪▪▪▪▪▪▪▪▪▪▪▪▪▪▪▪▪▪▪▪▪▪▪▪▪▪▪▪▪□□▪▪□▪▪▪▪▪▪	777777777763371	
1	▪□▪▪▪▪▪▪▪▪▪▪▪▪▪▪▪▪▪▪▪▪□▪□▪□□□□□□□□▪▪▪▪▪▪▪▪▪	577777752003771	
1	▪▪□▪▪▪▪▪▪▪▪▪▪▪▪▪▪▪▪▪▪▪□▪□▪□▪□□□□□□▪▪□▪▪▪▪▪▪	677777752403371	
1	▪▪▪▪▪▪▪▪▪▪▪▪▪▪▪▪▪▪□□□▪□□▪▪▪▪□□□□▪□▪▪□▪▪▪▪▪▪	777777047413371	
1	▪▪▪▪▪▪▪▪▪▪▪▪▪▪▪▪▪▪▪▪▪▪▪▪▪▪▪▪□▪▪□▪□□▪▪▪▪▪▪▪▪	777777777551771	
1	▪▪▪▪▪▪▪▪▪▪▪▪▪▪▪▪▪▪▪▪▪▪▪▪▪▪▪▪□□□□□□▪▪□▪▪▪▪▪▪	777777777403371	
1	▪▪▪▪▪▪▪▪▪▪▪▪▪▪□▪▪▪▪▪□▪▪▪▪▪▪▪□□□□▪□▪▪□▪▪▪▪▪▪	777767677413371	
1	□□□□□□□□□□□□▪▪□▪▪▪▪▪□▪▪▪▪▪▪□□□□□□□▪□□□□□□□□	000067677002000	
1	▪▪▪▪▪▪▪▪▪▪▪▪▪▪□▪▪▪▪▪▪▪▪▪▪▪▪□□▪▪▪▪□▪▪□□□□▪▪▪	777767777173031	
1	□□□□□□□□□□□□▪▪□▪▪▪▪▪□□□□□□□□□□□□□□□□□□□□□□□	000067600000000	
1	▪▪▪▪▪▪▪▪▪▪▪▪▪▪□▪▪▪▪▪▪▪▪▪▪▪□□□□□□□□▪▪□▪▪▪▪▪▪	777767776003371	
1	▪□□▪▪▪□▪▪▪▪▪▪▪□▪▪▪▪▪□▪▪▪▪▪▪□□□□□▪□▪□□□□▪▪▪▪	473767677012071	
1	▪▪▪▪▪▪□□▪▪▪▪▪▪□▪▪▪▪▪▪▪□▪▪▪▪▪□□□□□□▪▪□▪□▪▪▪▪	771767757403271	
1	▪□▪▪▪▪▪▪▪▪▪▪▪▪□▪□▪▪▪▪▪□▪▪▪▪▪□□□□▪□□▪▪▪▪▪▪▪▪	577765757411771	
1	▪▪▪▪▪▪▪▪▪▪▪▪▪▪▪▪▪▪▪▪▪▪▪▪▪▪▪▪□□□□▪□▪▪▪□▪□□□□	777777777413500	
1	□□□▪▪▪▪▪▪▪□▪▪▪□▪▪▪▪▪▪▪□▪▪▪▪□□□□□□□▪▪▪▪▪▪▪▪▪	077567757003771	
1	▪□□▪▪▪▪▪▪▪▪▪▪▪□▪▪▪▪▪□▪▪▪▪▪▪▪□□□□▪□▪▪▪▪▪▪▪▪▪	477767677413771	
1	▪▪▪▪▪▪□□▪▪▪▪▪▪□▪▪▪▪▪□□□□□□□□□□□□□□▪▪□▪□▪▪▪▪	771767600003271	
1	▪□▪▪▪▪▪▪▪▪▪▪▪▪□▪▪▪▪▪▪▪□▪▪▪▪▪□□□□▪□▪▪▪▪▪▪▪▪▪	577767757413771	
1	▪▪▪▪▪▪▪▪▪▪▪▪▪▪□▪▪▪▪▪□□□▪▪▪▪▪□□□□▪□▪▪□▪▪▪▪▪▪	777767617413371	
1	▪▪▪▪▪▪□□▪▪□□▪▪□▪▪▪▪□□▪□□▪▪□□□□□□□□▪▪□▪□▪▪▪▪	771467446003271	
1	▪▪▪▪▪▪□□▪▪□□▪▪□▪▪▪▪▪□▪□▪▪▪▪□□□□□□□▪▪▪▪□▪▪▪▪	771467657003671	
1	▪□▪▪▪▪▪▪▪▪▪▪▪▪▪▪▪▪▪▪▪▪□▪▪▪▪▪□□□□▪□▪▪▪▪▪▪▪▪▪	577777757413771	EAI6_BGD1 (882)
	**130"Modern" ** ***M. tuberculosis*** ** strains (TbD1 region deleted)**		
7	▪▪▪▪▪▪▪▪▪▪▪▪▪▪▪▪▪▪▪▪▪▪▪▪▪▪▪▪▪▪▪▪□□□□▪▪▪▪▪▪▪	777777777760771	T1 (53)
6	▪▪▪▪▪▪▪▪▪▪▪▪▪▪□▪▪▪▪▪▪▪▪▪▪▪▪▪▪▪▪▪□□□□▪▪▪▪▪▪▪	777767777760771	T2 (118)
6	▪▪▪□□▪▪▪▪▪▪▪▪▪▪▪▪▪▪▪▪▪▪▪▪▪▪▪▪▪▪▪□□□□▪▪▪▪▪▪▪	717777777760771	T1 (358)
5	▪▪▪□□□□▪▪▪▪▪▪▪▪▪▪▪▪▪▪▪□□□□□□□□□□□□□□▪▪▪▪▪▪▪	703777740000771	CAS (357)
6	▪▪▪▪▪▪▪▪▪▪▪▪▪▪▪▪▪▪▪▪□□□□▪▪▪▪▪▪▪▪□□□□▪▪▪▪▪▪▪	777777607760771	LAM9 (42)
5	▪▪▪▪▪▪▪▪▪▪▪▪▪▪▪▪▪▪▪▪▪▪▪▪▪▪▪▪▪▪▪▪□□□□▪▪□□□□▪	777777777760601	T1 (244)
36	□□□□□□□□□□□□□□□□□□□□□□□□□□□□□□□□□□▪▪▪▪▪▪▪▪▪	000000000003771	Beijing (1)
5	▪▪▪□□□□▪▪▪▪▪▪▪▪▪▪▪▪▪▪▪□□□□□□□□□□□□▪▪▪▪▪▪▪▪▪	703777740003771	CAS1_DELHI (26)
3	▪▪▪▪▪▪▪▪▪▪▪▪▪▪▪▪▪▪▪▪▪▪▪▪▪□□□□□□▪□□□□▪▪▪▪▪▪▪	777777774020771	H1( 47)
2	▪▪▪▪▪▪▪▪▪▪▪▪□▪▪▪▪▪▪▪▪▪▪▪▪▪▪▪▪▪▪▪□□□□▪▪▪▪▪▪▪	777737777760771	T3 (37)
2	▪▪▪▪▪▪▪▪▪▪▪▪▪▪▪▪▪▪▪▪▪▪▪▪▪▪▪▪▪▪▪▪□□▪▪▪▪▪▪▪▪▪	777777777763771	MANU2 (54)
1	▪▪▪▪▪▪▪▪▪▪▪▪▪▪▪▪▪▪▪▪▪▪▪▪▪▪□□▪▪▪▪□□□□▪▪▪▪▪▪▪	777777776360771	T1 (123)
1	▪▪▪▪▪▪▪□□□□□▪▪□▪▪▪▪▪▪▪▪▪▪▪▪▪▪▪▪▪□□□□▪▪▪▪▪▪▪	774067777760771	
1	▪▪▪□□□□□▪▪▪▪▪▪□▪▪▪▪▪□□□□□□□□□□□□□□▪▪▪▪□▪▪▪▪	701767600003671	
1	▪▪▪▪□□▪▪▪▪▪▪▪▪▪▪▪▪▪▪▪▪▪▪▪▪▪▪▪▪▪▪□□□□□□□□□□□	747777777760000	
1	▪▪▪▪□□□□▪▪▪▪▪▪▪▪▪▪▪▪▪▪▪▪▪▪▪▪▪▪▪▪□□□□▪▪▪▪▪▪▪	741777777760771	
1	□□□□□□□□□□□□□□□▪▪▪▪▪□▪▪□□□□□□□□□□□▪▪▪▪▪▪▪▪▪	000007660003771	
1	▪▪▪□□□□□▪▪▪▪▪▪□▪▪▪▪▪▪▪□□□□□□□□□□□□□□▪▪▪▪▪▪▪	701767740000771	
1	▪▪▪□□□□□□□□□▪▪□▪▪□▪▪▪▪□▪▪▪▪▪▪▪▪▪□□□□▪▪▪▪▪▪▪	700066757760771	
1	▪▪▪▪▪▪▪▪▪▪▪▪□▪□□▪▪▪▪▪▪▪▪▪▪▪▪▪▪▪▪□□□□▪▪▪▪▪▪▪	777723777760771	T3(1655)
1	▪▪▪▪▪▪▪▪▪▪▪▪▪▪□▪▪▪▪▪▪▪▪▪▪▪▪▪▪▪▪▪□□▪▪□□□□□□▪	777767777763001	
1	▪▪▪□□□□□▪▪▪▪▪▪□▪▪▪▪□□▪□□□□□□□□□□□□▪▪▪▪▪▪▪▪▪	701767440003771	
1	▪▪▪□□□□▪▪▪▪▪▪▪□▪▪▪▪▪▪▪□□□□□□□□□□□□▪▪▪▪▪▪▪▪▪	703767740003771	CAS1_DELHI (141)
1	▪▪▪□□□□▪▪▪▪▪▪▪▪▪▪▪▪▪▪▪▪▪▪▪▪▪□□□□□□▪▪▪▪▪▪▪▪▪	703777777403771	
1	▪▪▪□□▪□▪▪▪▪▪▪▪□▪▪▪▪▪▪▪□□▪▪▪▪▪▪▪▪□□▪▪▪▪▪▪▪▪▪	713767747763771	
1	▪▪▪□□▪▪▪▪▪▪▪▪▪□▪▪▪▪▪▪▪▪▪▪▪▪▪▪▪▪▪□□▪▪▪▪▪▪▪▪▪	717767777763771	
1	▪▪▪□□▪□□▪▪▪▪▪▪▪▪▪▪▪▪▪▪▪▪▪▪▪▪▪▪▪▪□□□□▪▪▪▪▪▪▪	711777777760771	
1	▪▪▪□□□□□□□□□▪▪□▪▪□▪▪▪▪▪▪▪▪▪▪▪▪▪▪□□▪▪▪▪▪▪▪▪▪	700066777763771	
1	▪▪▪▪▪▪▪▪▪▪▪▪▪▪▪▪▪▪▪▪▪▪▪▪▪▪▪▪▪▪▪▪▪□□□▪▪▪▪▪▪▪	777777777770771	CAS (1378)
1	▪▪▪▪▪▪▪▪▪▪▪▪▪▪▪▪▪▪▪▪□▪▪▪▪▪▪▪▪▪▪▪□□□□▪▪▪▪▪▪▪	777777677760771	T1 (291)
1	□□□□□□□□□□□□▪▪□▪▪▪▪▪□□□□▪▪□□□□□□□□▪▪▪▪▪▪▪▪▪	000067606003771	
1	▪▪▪□□□□▪▪▪▪▪▪▪▪▪▪▪▪▪▪▪▪□▪□□□□□□□□□□□▪▪▪▪▪▪▪	703777764000771	
1	▪▪▪▪▪▪▪▪▪▪▪▪▪▪□▪▪▪▪▪▪▪▪▪▪□□□□□□▪□□□□▪▪▪▪▪▪▪	777767774020771	H1 (151)
1	▪▪▪▪▪▪▪▪▪▪▪▪▪▪▪▪▪▪□▪▪▪□□▪▪▪▪▪▪▪▪□□□□▪▪▪▪▪▪▪	777777347760771	
1	▪▪▪□□□□▪▪▪▪▪▪▪▪▪▪▪▪▪□▪□□□□□□□□□□□□▪▪▪▪▪▪▪▪▪	703777640003771	
1	▪▪▪□□□□▪▪▪▪▪▪▪▪▪□▪□□□□□□□□□□□□□□□□□□▪▪▪▪▪▪▪	703775000000771	
1	▪▪▪□□□□□▪▪▪▪▪▪▪▪▪▪▪□□▪□□□□□□□□□□□□▪▪▪▪▪▪▪▪▪	701777440003771	
1	▪▪▪▪▪▪▪▪▪▪▪▪▪▪▪▪▪▪▪▪▪▪▪▪▪▪▪▪▪▪▪▪□□□□□▪▪▪▪▪▪	777777777760371	U (240)
1	▪▪▪□□▪□▪▪▪▪▪▪▪□▪▪▪▪▪▪▪▪▪▪▪▪□□□□□□□▪▪□□▪▪▪▪▪	713767777003171	
1	▪▪▪□□□□▪▪▪▪▪▪▪□▪▪▪▪▪▪▪▪▪▪▪▪□□□▪□□□□□▪▪▪▪▪▪▪	703767777040771	
1	▪▪▪□□▪▪▪▪▪▪▪▪▪□▪▪▪▪▪▪▪▪▪▪▪▪▪▪▪▪▪□□□□▪▪▪▪▪▪▪	717767777760771	
1	▪▪▪▪▪▪▪▪▪▪▪▪▪▪□□▪▪▪▪▪▪▪▪▪▪▪▪▪▪▪▪□□□□▪▪▪▪▪▪▪	777763777760771	T1 (732)
1	▪▪▪▪▪▪▪▪▪▪▪▪▪▪□▪▪▪▪▪□▪□▪▪▪▪▪□▪▪▪□□□□▪▪▪▪▪▪▪	777767657560771	
1	▪▪▪□□□□▪▪▪▪▪▪▪▪▪▪▪▪▪▪▪▪▪▪▪□□□□□□□□▪▪▪▪▪▪▪▪▪	703777776003771	
1	▪▪▪▪▪▪□□□□□□▪▪□▪▪□▪▪▪▪▪▪▪▪▪▪□▪▪▪□□□□▪▪□□□□▪	770066777560601	
1	▪▪▪□□□□▪▪▪▪▪▪▪▪▪▪▪▪▪▪▪▪▪▪▪▪▪□▪▪□□□▪▪□□▪▪▪▪▪	703777777543171	
1	□□□□□□□□▪▪▪▪▪▪□▪▪▪▪▪▪▪▪□▪▪▪□□□□□□□▪▪▪▪▪▪▪▪▪	001767767003771	
1	▪▪▪□□▪▪▪▪▪▪▪▪▪▪▪▪▪▪▪▪▪▪▪▪▪▪▪▪▪▪▪□□□□▪□□□□□▪	717777777760401	
1	▪▪▪□□□□▪▪▪▪▪▪▪▪▪▪▪▪▪▪▪▪▪▪▪▪▪▪▪▪▪□□▪▪▪▪▪▪▪▪▪	703777777763771	
1	□□□□□▪□□▪▪▪▪▪▪□▪▪▪▪▪▪▪▪▪▪▪▪▪□□□□□□▪▪▪▪▪▪▪▪▪	011767777403771	
1	□□□□□□□□▪▪□▪▪▪□▪▪▪▪▪□▪□▪▪▪▪□□□□□□□▪▪▪▪▪▪▪▪▪	001567657003771	
1	□□□□□□□□□□□□▪▪□▪□▪□□□□□□□□□□□□□□□□▪▪▪▪▪▪▪▪▪	000065000003771	
1	□□▪□□▪□□▪▪▪▪▪▪□▪▪▪▪▪□▪□▪▪▪▪▪□□□□▪□▪▪▪▪▪▪▪▪▪	111767657413771	
1	▪▪▪□□□□▪▪▪▪▪▪▪□▪▪▪▪▪▪▪□▪▪▪▪▪□□□□▪▪▪▪□□□▪▪▪▪	703767757417071	
1	□□□□□□□□□□□□□□□□□□□□□□□□▪▪▪▪□▪▪▪□□▪▪▪▪▪▪▪▪▪	000000007563771	
1	▪▪▪▪▪▪▪▪▪▪▪▪▪▪▪▪▪▪▪▪□□□□▪▪▪▪□▪▪▪□□□□▪▪▪▪▪▪▪	777777607560771	LAM6 (64)
1	□□□□□□□□□□□□▪□□▪▪▪▪▪□▪▪▪▪▪▪▪□▪▪□□□▪▪▪▪□□□□□	000047677543600	
1	▪▪▪□□▪▪▪▪▪▪▪▪▪□▪▪▪▪▪▪▪▪▪▪▪▪▪▪▪▪▪□□□□▪▪□▪▪▪▪	717767777760671	

**NOTE:** Filled square indicates “spacer present” in the DR region, non-filled square means “spacer deleted”.

* Number of isolates with the same spoligotype;

** Where shown, numbers in brackets indicate the “Shared-type number (SIT) of the SpolDB4/SITVIT database, in case the corresponding spoligotype was not represented in SpolDB4, the field was left empty.

All in vitro laboratory procedures were done at the Tuberculosis Laboratory of icddr,b at Dhaka. One spot sputum specimen was collected from each patient. Sputum AFB smear and culture were done on all the collected specimens and only those found to be culture positive were included in the study. Concentrated sputum smears were examined for AFB using the Ziehl-Neelsen staining under light microscope. Sputum specimens were decontaminated following the Petroffs’ NaOH method [15]. The supernatant was discarded and 2 loopfuls of palette were inoculated on 2 Lowenstein Jensen (L-J) slants. These slants were incubated at 37°C for 8 weeks and the inoculated slants were examined once every week for contamination as well as growth of visible mycobacterial colony. Sputum was considered culture negative when no visible mycobacterial colony was grown on either of the L-J slants within 8 weeks of incubation at 37°C.

The standard proportion method of Canetti et al. was followed to test for susceptibility of *M. tuberculosis* isolates to INH (0.2 mg/l), RMP (40 mg/l), ETM (2 mg/l) and STM (4 mg/l) [16]. Isolate was considered resistant to a given drug when any growth of 1% or more above the control was observed in each drug- containing quadrant plate. DST reports of the patients were provided to the health facility to ensure the management according to the sensitivity profile.

Genomic DNA was extracted by re-suspending mycobacterial colonies in 100–200 µl of distilled H_2_O and incubating at 85°C for 30 min. After centrifugation of the suspension, the supernatant containing the DNA was taken and stored at –20°C until further use. PCR analysis was done using previously described methods [9]. Sequences inside or flanking RD9 and TbD1 regions were obtained from the web sites http://genolist.pasteur.fr/TubercuList/and http://www.sanger.ac.uk/Projects/M_bovis/. Primers were designed by using the Primer3 web site http://www-genome.wi.mit.edu/cgi-bin/primer3-www.cgi that would amplify ∼500 bp fragments. PCRs were performed on a PTC-200 amplifier (MJ Inc.) and run on agarose gel. In all PCR, DNA from H37RV and known ancestral and modern *M. tuberculosis* strains were included as the control.

Spoligotyping was performed as previously described by Kamerbeek et al. [11] with minor modifications. The direct repeat (DR) region was amplified by PCR with oligonucleotide primers derived from the DR sequence. Mycobacterial genomic DNA was extracted from cultured cells as described previously [17], [18], [19]. Twenty-five microlitres of the following reaction mixture were used for the PCR: 12.5 µl HotStarTaq Master Mix (Qiagen; this solution provides a final concentration of 1.5 mM MgCl2 and 200 µM each dNTP), 2 µl each primer (20 pmol each), 5 µl DNA solution (approx. 10 ng), and 3.5 µl distilled water). The amplified product was hybridized to a set of 43 immobilized oligonucleotides, each corresponding to one of the unique spacer DNA sequences within the DR locus. Detection of hybridizing DNA was by the chemiluminescent ECL method (Amersham) [9], [19]and by exposure to X-ray film (Hyperfilm ECL: Amersham) in accordance with the instructions of the manufacturer.

Data were entered using the software package SPSS 17.0 and checked for errors. Pearson’s chi-square test was used to determine statistical associations between isolates and drug resistance patterns with SPSS software. A *P* value of <0.05 was considered evidence of a significant difference.

## Results

During the study period a total of 265 smear positive PTB patients were enrolled and interviewed. Out of the 265 patients, sputum samples from 197 (74%) were positive on culture and 68 (26%) were negative on culture. However, among the 197 culture-positive cases 189 isolates were identified as *M. tuberculosis* complex whereas the remaining 8 of the isolates were found to be nontuberculous mycobacteria (NTM). Thus, anti-TB drug susceptibility results from a total of 189 isolates were included in the current study (Figure 1). Geographical locations were available for 171 patients. Among them half of the patients were from Dhaka division, the remaining 39(23%), 18(11%), 11(6%), 3(2%), 7(4%), and 9(5%) were from Chittagong, Barisal, Khulna, Rangpur, Sylhet and Rajshahi divisions respectively [Table 1] (Figure 2).

Characteristics of the patients as shown in Table 2; 142(75%) were males and 47(25%) were females, with a male to female ratio of 3∶1. The mean age of these patients was 32.8 years (range: 12–70). Seventy eight percent of the patients were below 45 years of age. Among the available data, 10% of the patients had history of exposure to TB patient within the family and an additional 34% had exposure to TB patients other than family members. Eighty-eight percent (n = 167) had a history of previous anti-TB treatment while the remaining 12% had never received anti TB treatment. Forty one percent of 161 patients had history of completion of anti-TB treatment and 59% did not complete the treatment previously.

As shown in Table 3, the overall drug susceptibility patterns showed that of the 189 subjects investigated, 172(91%) were infected with *M. tuberculosis* strains that were resistant to at least one of the four first-line anti-TB drugs tested, and MDR was detected in 139(74%) isolates. Resistance to STM was observed in 149(79%), to INH in 150(79%), to RMP in 146(77%), and 145(77%) cases showed resistance to ETM. Overall mono-resistance was observed as follows: against STM in 10(5%), INH in 1(0.5%), against RMP in 2(1%) and against ETM in 4(2%) subjects. Alarmingly, 122(65%) isolates were found to be resistant to all four anti-TB drugs. MDR was significantly higher among the patients with history of anti-TB treatment previously (95% confidence interval, p = 0.001). MDR TB was also high among males (75%) and in patients with age below 45 years (76%), which was not statistically significant. MDR TB cases were almost equally distributed in all the divisions except Rangpur where all three TB cases were found to be MDR. There was no significant difference between MDR and non-MDR strain distribution among the divisions.

The genomic region of difference 9 (RD9) is strictly conserved in *M. tuberculosis* and absent from the *Mycobacterium africanum* - *Mycobacterium bovis* within the *M. tuberculosis* complex [9]. In all 189 isolates this region was present and the isolates thus confirmed as *M. tuberculosis*. The presence of the TbD1 region was observed in 59 strains (31%) indicating that these strains belong to the “ancestral” *M. tuberculosis* type while 130 (69%) strains had the TbD1 region deleted and thus belonged to the “modern” type (Table 4). Modern strains were found to be higher among patients with age <45 years (p<0.03). Males comprise of 48(34%) ancestral strains and 94(66%) modern strains. Among previously treated TB cases ancestral and modern strains were 53(32%) and 114(68%) respectively. Highest levels of ancestral strains (33) were found in Dhaka division, whereas 8 and 6 were found in Chittagong and Sylhet divisions respectively. In other divisions the number of ancestral strains was low. Similarly the highest number of modern strains (51) was found in Dhaka division. Interestingly ancestral strains were predominant in Sylhet division and in all other divisions modern strains predominated (Table 1). A univariate analysis showed that a significant difference (p = .006) was found between presence of ancestral and modern strains.

Spoligotyping is a rapid and convenient method that ensures the identification of clades of strains. To determine the strain lineage present in the sample, 189 isolates were spoligotyped and the binary outcomes compared with those existing in SpolDB4 database. In the present study, typing of 189 strains resulted in 105 different spoligotype patterns. One hundred strains were grouped into 16 clusters and 89 (47%) strains displayed unique profile. A cluster was defined as two or more strains from different patients with identical spoligotype patterns, on the other hand non-clustered patterns were referred to as unique. As shown in Table 4, the largest cluster comprised 36 (19%) strains, which were referred to as ‘Beijing’ family (octal spoligotype code 000000000003771) and this group was found most predominant. Interestingly, 27 (75%) strains of the ‘Beijing’ family were found to be MDR (not statistically significant). Thirty (16%) of the isolates were of T type which corresponds to a group of TbD1^−^ *M. tuberculosis* strains that mainly showed octal patterns 777777777760771, 777767777760771, 717777777760771 or 777777777760601 (Table 4). Among the ancestral TbD1+ *M. tuberculosis* strains eighteen isolates showed patterns belonging to the East African Indian (EAI) spoligotypes represented in SpolDB4, including EAI5, EAI1_SOM, EAI3_InD, EAI6_BGD1, EAI undefined. The characteristics of EAI spoligotypes are the presence of spacer 33, as well as the absence of spacers 28–32, and spacer 31. The remaining clusters contained 2 to 6 strains in each group, which include Central Asian strain type 1 (CAS1 or Dehli), LAM, MANU2, H and U families.

## Discussion

MDR-TB usually develops during treatment of fully sensitive TB when the course of antibiotics is interrupted and concentrations of active drugs in the body are insufficient to kill 100% of bacteria. This can happen for a number of reasons: Patients may feel better and halt their antibiotic course, drug supplies may run out or become scarce, or patients may forget to take their medication from time to time. Although mutations that cause resistance to certain drugs may also cause lower fitness in the mutated *M. tuberculosis* strains, selected mutation sites may allow MDR *M. tuberculosis* strains to spread from person to person as readily as drug-sensitive TB and in the same manner [20].

The results of our study show a high level drug resistance among the admitted patients of the tertiary reference hospital, the National Institute of Diseases of the Chest and Hospital situated in Dhaka city. The results are comparable with the results of the study involving randomly selected patients having clinical and/or radiological features of tuberculosis attending the same hospital [21], [22]. Among 363 cases, resistance rates for INH, RMP, EMB and STM were 76.3%, 71.6%, 27.6% and 55.7% respectively with a total of 221(60.9%) cases detected as MDR-TB [22]. Our study also shows very high level of MDR cases (73.5%) among the referred cases attending this tertiary institution. Global report on multidrug and extensively drug-resistant TB surveillance and response by WHO in 2010 highlighted the results of a population based case study by the Damian Foundation, Bangladesh, which showed that 28% of the 599 previously treated cases notified in 2008 had confirmed MDR-TB, with particularly high risk of MDR-TB among cases failing treatment [23]. Among cases that failed an initial treatment regimen, 58% had MDR-TB. Among those that failed a Category II retreatment regimen, 91% had MDR-TB [8]. This indicates the higher degree of drug resistance among patients who received anti-TB treatment previously or those having chronic TB. In previously treated patients, the chances of resistance to any drug is over 4-fold higher, and of MDR-TB over 10-fold higher, in comparison to untreated patients [24]. The number of previously treated cases in the country correlate with the overall prevalence of drug resistance, as drug resistance is more commonly found in cases with previous history of anti-TB medication. Cases with previous treatment ranged from 4.4% to 26.9% of all registered patients under DOTS programmes in countries with a high burden of TB [24]. In case of China and India, the two largest high-TB burden countries, 20% of sputum smear-positive cases had prior history of treatment [25]. Retrospective chart review based on positive cultures isolated in a high volume mycobacteriology laboratory in Christian Medical College Vellore, India between 2002 and 2007 examined 47 XDR, 30 MDR and 117 susceptible controls. Drug resistant cases were less likely to be extrapulmonary, and mostly had received previous treatment regimens [26].

In this study almost half of the study subjects were from the Dhaka division, the number of MDR cases was higher in this location than other areas of Bangladesh. The results of our study showed that males were more commonly infected with TB (75%), including MDR-TB (75%) than females. This trend is also observed in some other studies [27], [28]. In Western Europe MDR-TB cases were more common in males while, there was no association of MDR-TB with the male gender in Eastern Europe [29], [30]. One of the underlying causes for high rate of MDR-TB among males is believed to be the reduced adherence of males to treatment compared to females. Another important finding of our study was the higher incidence of MDR-TB among the patients below the age of 45 years (76%). This finding has also been observed in other studies [30], [31], [32]. This is possibly due to the exposure of elderly persons to the organisms in the past, when the circulating bacilli were susceptible, and during the process of treatment, resistance was acquired while young patients are more likely to have acquired the bacilli more recently when they were more likely to be resistant. However, to confirm the findings of this study it is important to perform a molecular epidemiological study with a larger sample size.

In this study the strains were also characterized by deletion analysis and spoligotyping. Deletion analysis using RD9 revealed that all the samples investigated were *M. tuberculosis*. Screening for TbD1, the ‘*M*. *tuberculosis* specific deletion region 1′ was done for all the samples. The TbD1 analysis employed in this study showed that the majority of the isolates (69%) belonged to the TbD1- modern strains, whereas this region was found intact among 59 (31%) samples and these strains were named ancestral type *M. tuberculosis* strains because they belong to a lineage of strains that divided from all other *M. tuberculosis* strains before the deletion of TbD1- occurred [9], [33]. The percentage of modern strain was higher in this study than the study conducted in rural area of Bangladesh [34]. Multi drug resistance was significantly higher among the modern type strains than the ‘ancestral’ strains (data not shown).

In our study the most predominant spoligotype of strains was the Beijing genotype, it represented about 19% (36 strains) of all isolates. The majority of the Beijing family members originated from the province of Beijing in China, and strains of this family are highly prevalent in many Asian countries [35], [36]. Rate of infection with Beijing family strains are higher in Asia than those in the more distant countries, suggesting that the Beijing family may have radiated from the Beijing area to other regions. Almost all of the Beijing isolates were from the patients who were previously treated with antitubercular drugs for certain periods. Previous data showed that Beijing strains are often associated with drug resistant TB [37], [38], [39], [40]. Majority of the Beijing strains (75%) were found to be MDR in this study. Previous study showed that 31% strains were of Beijing type which is higher than what has been found in this study [33]. In addition to the prevalent Beijing family strains, the second most frequently occurring strains showed spoligotype characteristic of the “T” family. Among the T family, T1 was most predominant (70%) in which spacer 33 to 37 along with spacer 4, 5 were absent. Among the T family 21 isolates were MDR. Although the T family is one of the most prevalent types, it remains an ill-defined family of *M. tuberculosis* that is found worldwide [41], [42]. It has been suggested from our study findings that strains of this family are also prevalent in Bangladesh. Our results suggest that emergence of drug resistant *M. tuberculosis* strains belonging to the ‘Beijing’ and the ‘T’-types remains a serious threat to the local TB control program.

At the time of planning of this study we didn’t have an accurate idea of drug resistance nationally. As the NIDCH had patients from all over the country, the intention was to get deeper insights into the drug sensitivity profile and the strain pattern in a cohort of patients admitted to this tertiary referral hospital. In our NTP treatment strategy, patients with PTB are usually treated in outpatient department (DOTS corner) unless the patient is seriously ill or associated with other complications or non responsive to the applied chemotherapy. According to the national TB control guideline suspected MDR-TB cases from all over the country are usually referred to NIDCH. Thus, the patients visiting this health facility are representative of the country as a whole. However, it should be mentioned that among the enrolled patients, re-treatment cases and chronic TB cases were frequent, which might have contributed towards the relatively high number of patients with drug resistant TB identified, that therefore may not reflect the community status.

In summary, the drug-resistance rate of PTB, especially MDR-TB, was higher in patients with previously incomplete anti-tuberculosis treatment at a tertiary referral hospital in Bangladesh. A high level of drug resistance among the re-treatment TB patients poses a threat of transmission of resistant strains to susceptible persons in the community. For these reasons molecular characterization and determination of individual drug resistance of patient isolates is of importance. Proper counseling of patients and attention towards the completion of the anti-TB treatment are needed. In TB prevalent areas, more studies on anti-TB drug resistance preferably population based continuous surveillance should be carried out.
